# Global prevalence and risk factors of *Cryptosporidium* infection in *Equus*: A systematic review and meta-analysis

**DOI:** 10.3389/fcimb.2022.1072385

**Published:** 2022-11-25

**Authors:** Xiao-Man Li, Hong-Li Geng, Yong-Jie Wei, Wei-Lan Yan, Jing Liu, Xin-Yu Wei, Miao Zhang, Xiang-Yu Wang, Xiao-Xuan Zhang, Gang Liu

**Affiliations:** ^1^ College of Veterinary Medicine, Qingdao Agricultural University, Qingdao, Shandong, China; ^2^ College of Life Science, Changchun Sci-Tech University, Shuangyang, Jilin, China; ^3^ College of Animal Science and Veterinary Medicine, Heilongjiang Bayi Agricultural University, Daqing, Heilongjiang, China

**Keywords:** *Cryptosporidium*, *Equus*, meta-analysis, prevalence, zoonotic diseases

## Abstract

**Intoduction:**

Cryptosporidiosis is a zoonotic disease caused by Cryptosporidium infection with the main symptom of diarrhea. The present study performed a metaanalysis to determine the global prevalence of Cryptosporidium in Equus animals.

**Methods:**

Data collection was carried out using Chinese National Knowledge Infrastructure (CNKI), VIP Chinese journal database (VIP), WanFang Data, PubMed, and ScienceDirect databases, with 35 articles published before 2021 being included in this systematic analysis. This study analyzed the research data through subgroup analysis and univariate regression analysis to reveal the factors leading to high prevalence. We applied a random effects model (REM) to the metadata.

**Results:**

The total prevalence rate of Cryptosporidium in Equus was estimated to be 7.59% from the selected articles. The prevalence of Cryptosporidium in female Equus was 2.60%. The prevalence of Cryptosporidium in Equus under 1-year-old was 11.06%, which was higher than that of Equus over 1-year-old (2.52%). In the experimental method groups, the positive rate detected by microscopy was the highest (10.52%). The highest Cryptosporidium prevalence was found in scale breeding Equus (7.86%). The horses had the lowest Cryptosporidium prevalence (7.32%) among host groups. C. muris was the most frequently detected genotype in the samples (53.55%). In the groups of geographical factors, the prevalence rate of Cryptosporidium in Equus was higher in regions with low altitude (6.88%), rainy (15.63%), humid (22.69%), and tropical climates (16.46%).

**Discussion:**

The search strategy use of five databases might have caused the omission of some researches. This metaanalysis systematically presented the global prevalence and potential risk factors of Cryptosporidium infection in Equus. The farmers should strengthen the management of young and female Equus animals, improve water filtration systems, reduce stocking densities, and harmless treatment of livestock manure.

## Introduction

*Cryptosporidium* is a zoonotic coccidian parasite, which mainly parasitizes in the epithelial cells of the small intestine in vertebrates (Xue, 2021). The life cycle of *Cryptosporidium* in hosts comprises asexual and sexual phases, and finally, the oocysts were shed with faeces. The oocyst containing infective sporozoites can survive for months in moist conditions (Chalmers et al., 2019). The *Cryptosporidium*-infected patients usually display signs of diarrhea and abdominal pain, and other clinical features include nausea, vomiting, and low-grade fever. The occasional signs include myalgia, weakness, malaise, headache, and anorexia. The severity, persistence, and eventual outcome of infection generally depend on the characteristics of *Cryptosporidium* and host factors (Bouzid et al., 2013).

*Cryptosporidium* was examined in mucosal tissues of mice by Tyzzer in 1907 (Zhang and Jiang, 2001). *Cryptosporidium* was the first detection happened to be in immunodeficient Arabian horse foals in 1978 (Snyder et al., 1978). People didn’t pay attention to the disease at first. At the end of the nineteenth century, the events of death of AIDS patients who were infected with *Cryptosporidium* caused a full attention of cryptosporidiosis (Current et al., 1983). *Cryptosporidium* is worldwide distributed, and the infections of *Cryptosporidium* in organisms have been reported in more than 70 countries (Xue, 2021).

*Equus* animals are mainly distributed in Eurasia and Africa, roughly divided into three species: horse, donkey and zebra. Horses are now mainly used for entertainment or competition, donkeys are kept as companions or used as working or production animals, whereas mules are mainly used for working purposes (Jian, 2012). Because they are maintained in a close association with their owners and veterinary personnel, *Equus* animals are the important reservoirs for transmission of pathogens (such as *Cryptosporidium hominis* and *Toxoplasma gondii*) to humans and other animals (Alvarado-Esquivel et al., 2015; Jian et al., 2016). The cases of *Equus* cryptosporidiosis have been reported in many countries around the world, such as China, Italy, and United States. (Veronesi et al., 2010; Qi et al., 2015; Wagnerová et al., 2016).

At present, the systematic evaluation and analysis of *Equus* cryptosporidiosis are still absent. Therefore, a systematic review and meta-analysis were conducted to evaluate the prevalence of *Equus* cryptosporidiosis in the world. The collected information was used to discuss the factors affecting the infection of *Cryptosporidium* in *Equus* (Page et al., 2021).

## Materials and methods

### Search strategy

**Figure 1:**
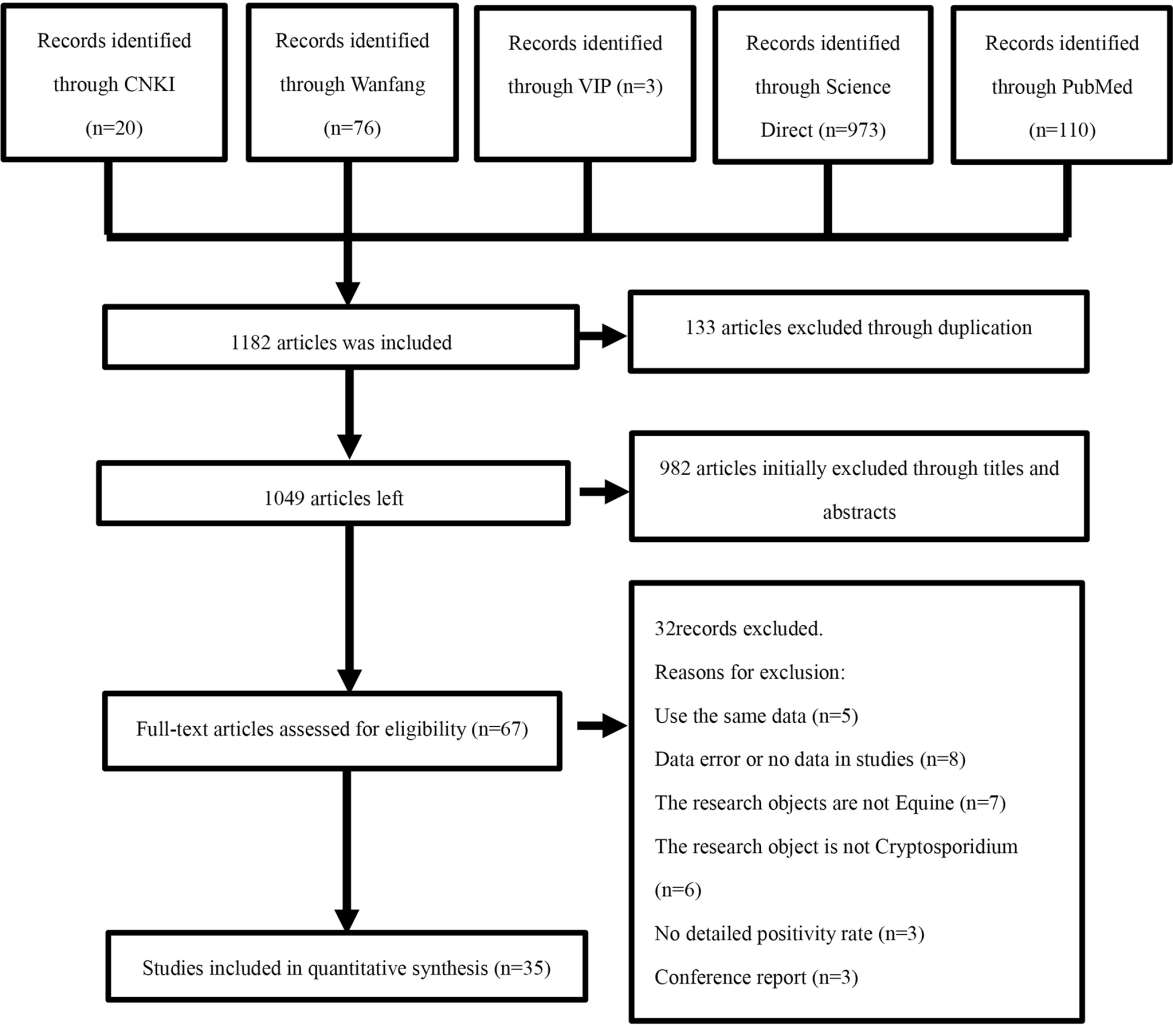
Flow diagram of literature search and selection.

To evaluate the prevalence of *Cryptosporidium* infections in *Equus* around the world, we performed a comprehensive review of literatures both in Chinese and English published from the beginning of the creation to October 1, 2021. The articles were derived from five databases, including CNKI, VIP Chinese Journal Database, Wanfang Data, PubMed, and ScienceDirect. In the three Chinese databases, the advanced search was carried out using “*Equus* (in Chinese)” and “*Cryptosporidium* (in Chinese)” as keywords. In Science Direct, the keywords “*Cryptosporidium*” and “*Equus*” were used for a search. We used MeSH terms “*Cryptosporidium*” and “Equine” and their entry terms, such as “Horse”, “Horse, Domestic”, “Domestic Horse”, “Domestic Horses”, “Horses, Domestic”, “*Equus*” “caballus”, and “*Equus* przewalskii” in PubMed. We used boolean operators “AND” to connect MeSH terms and “OR” to connect the entry terms. Finally, the search formula was “((*Cryptosporidium*) OR *Cryptosporidiums*) AND ((((((((Horse) OR Horse, Domestic) OR Domestic Horse) OR Horses, Domestic) OR *Equus* caballus) OR *Equus* przewalskii) OR Horse genus) “. Endnote (X9.2 version) was employed to organize the obtained article information. A protocol for the literature review was devised (**Figure 1**) in accordance with the PRISMA guidelines.

### Inclusion and exclusion criteria

As part of the eligibility for inclusion, titles that suggested the topic *Cryptosporidium* in *Equus* were selected. The abstracts from the selected reference titles were reviewed by two independent reviewers to determine if the studies met the inclusion criteria and, if so, the entire articles were reviewed in full. The inclusion criteria for the systematic review and meta-analysis were as follows: (1) the object of research was *Equus*; (2) the diseased individuals were positive for *Cryptosporidium* in the research; (3) the research contained clear information, including the number of sick individuals and the population, the number of positive samples, the location of the test, and the location of sampling; (4) the article should contain a full text; (5) the research must be designed for a cross-sectional extension. Articles that do not meet these criteria were excluded. Unpublished reports, comments and copies were also excluded.

### Data extraction

The extracted data included article title, first author, publication year, detection method, breeding environment, breeding method, genotypes, sampling year, article quality, detailed geographic and climatic factors, total number, number of positives, age and gender of the research object, geographic location (latitude and longitude), altitude, relative humidity, annual average temperature, annual precipitation, climate, and detection method type. The meteorological data of the years involved were from the China Meteorological Data Service Center (CMDC, http://data.cma.cn/) and national centers of environmental information (https://www.ncei.noaa.gov/maps/monthly/), such as temperature, rainfall, longitude, latitude, humidity and altitude. The database was established by using Microsoft Excel (version 16.32). Two authors independently extracted and recorded data from each selected study. The differences derived from reviewers or uncertainty about the qualifications of the research were further assessed by another author of this paper. The grouping method is based on previous studies (Wei et al., 2021a; Wei et al., 2021b).

### Quality assessment

The quality of the included studies was evaluated according to the criteria based on the recommended grading evaluation, formulation, and the Grading of Recommendations Assessment, Development and Evaluation (GRADE) (Xie and Machado, 2021). The scoring criteria were as follows: (1) whether a clear detection method was employed; (2) whether the sampling animal was clear; (3) whether three or more influencing factors were included; (4) the sample size was greater than enough; and (5) whether the sampling year was clear. Therefore, the studies could be scored between 0 and 5 points.

### Statistical analyses

The meta package in R software version 4.0.3 (“R core team, R: A language and environment for statistical computing” R core team 2018) was employed to analyze the data in this study (Zeng et al., 2020). Before performing the meta-analysis, we tested five transformation methods to bring the data closer to a Gaussian distribution, namely no transformation (PRAW), logarithmic transformation (PLN), logit transform (PLOGIT), arcsine transform (PAS), and double arcsine transform (PFT) (**Table 1**). The conversion rate was based on a Shapiro-Wilk normal test. The W-value close to 1 and the *P*-value greater than 0.05 was close to the Gaussian distribution criterion. The heterogeneity between studies was calculated by *Cochran*-Q, *I^2^* statistics, and *χ* ^2^ test, the *P*-value < 0.05 and *I^2^* = 50% was used to define the degree of heterogeneity with a statistical significance. According to the heterogeneity of the included articles, a random effect model was selected for analysis (Ni et al., 2020). The forest plots were used for a comprehensive analysis. The funnel plot and Egger’s test were used to evaluate publication bias of studies. When there is publication bias in the included articles, the funnel plot is asymmetric, and the distribution is skewed (Egger et al., 1997). The Egger’s test is expected to have a regression intercept of 0 in the absence of bias (Lin and Chu, 2018). The stability of a study was evaluated by the trim and fill analysis and sensitivity analysis (Wang et al., 2021).

**Table 1:** Normal distribution tests for normal rates and different transitions.

Conversion form	W	P
PRAW	0.8277	7.482e-05
PLN	NaN	NA
PLOGIT	NaN	NA
PAS	0.94712	0.09241
PFT	0.94693	0.09118

“PRAW”: raw exchange rate; PLN: log conversion. “PLOGIT”: logit transformation; “PAS”: arcsine transformation; “PFT”: double arcsine transformation; “NaN”: meaningless number; NA: data is missing.

To further study the potential sources of heterogeneity, the individual and multivariate model factors were analyzed to determine factors that affected heterogeneity. The factors included sampling surveys (before 2008 vs. others), diagnostic methods (molecular diagnostics vs. other methods), age ≤ 1year vs. age >1year, genotype (*C. parvum* vs. others), gender (female vs. male), feeding method (cage-free vs. scale breeding), host (horse vs. others), country (China vs. others), the quality level of publications (5 points vs. others), longitude (<50° vs. others), latitude (<30° vs. others), annual average rainfall (< 500 mm vs. ≥ 500 mm), annual average temperature (< 10°C vs. ≥ 10°C), annual average humidity (40%-50% vs. others), altitude (<50 m vs. others), and climate (temperate climate vs. others)

## Results

### Search results

In this study, 1,182 related articles were identified after searching five databases, and 67 articles were selected after the initial screening and removal of duplicates. According to the selection criteria described in section, an additional 32 indeterminate articles were excluded after checking the full text. Finally, 35 articles were selected for the meta-analysis. (**Figure 1**; **Table 2**).

**Table 2:** Studies in the analysis.

Study ID	Sampling time	Country	Test method	Event	Positive rate
Xiao L et al. (1994)	1992.3-1992.10	America	Direct immunofluorescence staining method	16/222	0.072
Johnson et al. (1997)	1994.8-1994.10	America	Direct fluorescent antibody	0/91	0.000
Olson et al. (1997)	1996.7	Canada	Microscopic examination	6/35	0.171
Majewska et al. (1999)	NA	Poland	Enzyme immunoassay	10/106	0.094
Majewska et al. (2004)	NA	Poland	Microscopic examination	11/318	0.035
Grinberg et al. (2009)	2005-2007	New Zealand	Microscopic examination	12/67	0.179
De Souza et al. (2009)	NA	Brazil	Microscopic examination	3/396	0.008
Veronesi et al. (2010)	2007.2-4	Italy	DFA	12/150	0.080
Burton et al. (2010)	2009.2-5	America	DFA	16/349	0.046
Perrucci et al. (2011)	2006-2008	NA	ELISA	2/74	0.027
Jian (2012)	2008.7-2013.9	China	Microscopic examination	222/1302	0.171
Inácio et al. (2012)	2010.11-2011.3	NA	Microscopic examination	39/196	0.199
Caffara et al. (2013)	NA	Italy	PCR	14/37	0.378
Laatamna et al. (2013)	2010-2011	Algeria	PCR	4/138	0.029
Guo P et al. (2014)	2001.9-2003.10	China	PCR	161/436	0.369
Qi et al. (2015)	2013.8-9	China	PCR	7/262	0.027
Liu A et al. (2015)	NA	China	PCR	3/29	0.103
Laatamna et al. (2015)	2011.11-2013.5	Algeria	PCR	7/343	0.020
Kostopoulou et al. (2015)	NA	Belgium et al.	PCR	8/398	0.020
Zhou H. (2015)	2013.3-2014.5	China	Microscopic examination	30/508	0.059
Galuppi et al. (2015)	2011.12-2012.12	Italy	PCR	14/73	0.192
Wagnerová et al. (2015)	2011-2012	NA	PCR	12/352	0.034
Wagnerová et al. (2016)	NA	America	PCR	28/84	0.333
Hijjawi et al. (2016)	2014.10-2015.5	NA	PCR	6/74	0.081
Song Y et al. (2017)	NA	China	PCR	1/10	0.100
Deng L et al. (2017)	2015.8-2016.4	NA	PCR	6/333	0.018
Inácio et al. (2017)	2010.11-2011.3	Brazil	PCR	20/92	0.217
Raue et al. (2017)	2004-2012	Germany	Microscopic examination	4/21	0.190
Deng (2018)	2015.7-2017.5	China	PCR	6/441	0.014
Zhang Q et al. (2019)	2018.5-7	China	PCR	0/32	0.000
Li F et al. (2019)	2015-2019	China	PCR	90/878	0.103
Wei Z. (2020)	2016.2018.6	China	PCR	11/621	0.018
Couso-Pérez et al. (2020)	NA	Portugal,Spain	IFAT	10/79	0.127
Wang (2020)	2016.2-2018.12	China	Microscopic examination	16/680	0.024
Zhang (2021)	2018-2020	China	PCR	9/590	0.015

NA, data is missing; PCR, Polymerase Chain Reaction; IFAT, International Federationt for Alternative Trade; DFA, Direct Immunofluorescence Assay; ELISA, Enzyme-linked Immunosorbent Assay.

### Qualification research and publication bias

The included articles involved 13 countries. In the 35 studies, the total number of samples was 9,817, and the number of positives was 816 (**Table 3**). According to our quality criteria, 11 articles were considered to be 5 points, 21 were of medium-quality (three or four points), and the remaining 3 articles were deemed to be of low-quality (zero to two points; **Table 3**).

**Table 3:** Summary of global equine *Cryptosporidium* infection rates.

Variable	Category	No. studies	No. examined	No. positive	% (95% CI*)	Heterogeneity			Univariate meta-regression	
						*χ* ^2^	*P-*value	*I* ^2^ (%)	*P-*value*	Coefficient (95% CI)
Detection methods	Molecular diagnostics	21	5982	430	7.49% (3.77-12.35)	603.44	< 0.01	96.7	0.3555	-0.0679(-0.2121 to 0.0762)
	Microscopic examination	8	2843	327	10.52% (4.99-17.79)	200.33	< 0.01	96.5		
	Immunological detection	7	1071	59	4.37% (1.83-7.95)	27.32	< 0.01	78.0		
Gender	Female	12	1564	52	2.60% (0.78-5.44)	61.96	< 0.01	82.2	0.8605	-0.0124 (-0.1506 to 0.1258)
	Male	6	452	12	2.36% (0.04-8.07)	21.05	< 0.01	76.2		
Year	≤1year	19	3257	394	11.06% (6.13-17.22)	346.09	< 0.01	94.8	0.0014	-0.1792(-0.2889 to -0.0695)
	> 1year	16	3602	131	2.52% (0.97-4.77)	156.63	< 0.01	90.4		
Geographic area	Asia	14	5088	366	5.44% (2.24-9.94)	465.79	< 0.01	97.2	0.1730	0.2025(-0.0888 to 0.4938)
	Oceania	1	67	12	17.91% (9.73-27.92)	0.00	NA	NA		
	Europe	9	1534	95	10.15% (4.77-17.24)	74.19	< 0.01	89.2		
	Africa	2	481	11	2.27% (1.13-3.79)	0.30	0.58	0.00		
	North America	4	746	60	7.37% (0.08-24.78)	69.68	< 0.01	95.7		
	South America	4	719	68	12.45% (2.84 -27.51)	101.94	< 0.01	97.1		
Sampling year	Before 2008	9	1361	241	10.77% (3.99-20.32)	214.05	< 0.01	96.3	0.0125	-0.1615 (-0.2883 to -0.0348
	2009-2012	10	2580	306	10.73% (5.79-16.95)	200.18	< 0.01	95.5		
	After or 2013	10	4419	181	3.04% (1.52-5.06)	114.57	< 0.01	92.1		
Feeding method	Cage-free	3	555	48	5.09% (0.00–20.00)	64.51	< 0.01	96.9	0.5752	-0.0584(-0.2628 to 0.1459)
	Scale breeding	18	4995	546	7.86% (4.39-12.22)	500.57	< 0.01	96.6		
Host	Zebra	1	10	1	10.00% (0.01-34.87)	0.00	NA	NA	0.6247	0.1419(-0.4267 to 0.7105)
	Horse	32	6851	480	7.32% (4.49-10.78)	684.18	< 0.01	95.5		
	Mule	1	50	10	20.00% (10.20-32.09)	0.00	NA	NA		
	Donkey	5	2906	333	7.52% (2.25-15.54)	196.58	< 0.01	98.0		
Fraction	middle	21	3138	193	7.44% (4.05-11.74)	224.33	< 0.01	91.1	0.0939	0.1839 (-0.0313 to 0.3990)
	high	11	6410	575	5.93% (2.17-11.37)	606.89	< 0.01	98.4		
	low	3	269	48	17.36% (5.92-33.15)	18.76	< 0.01	89.3		
Total		35	9817	816	7.59% (4.86-10.87)					

CI*: confidence interval; NA*: not applicable; P value *: P < 0.05 was statistically significant.

Quality *: High: 5 points; Medium: 3 or 4; Low: 2.

**Figure 2:**
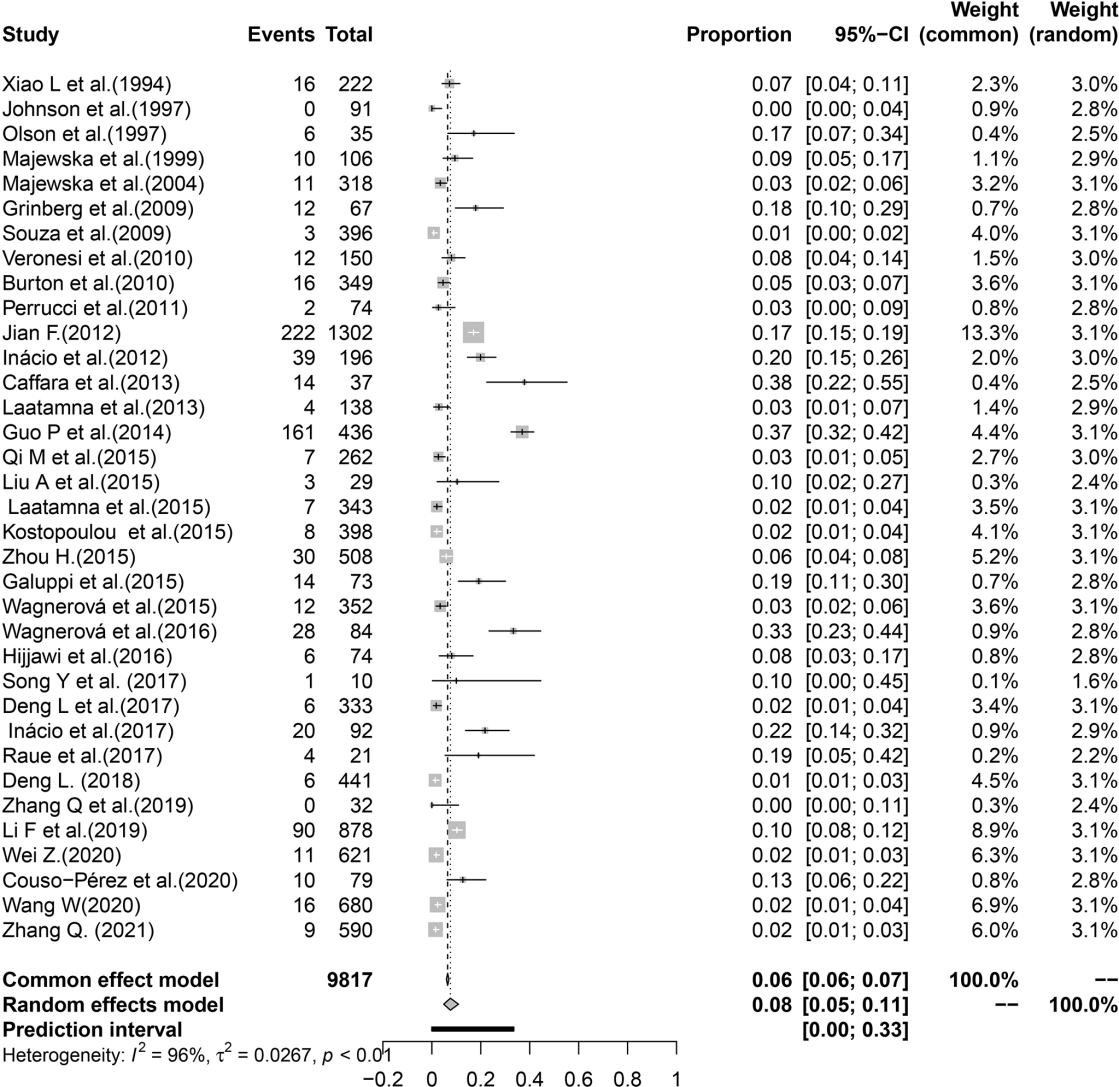
Forest map of global cryptosporidiosis in *Equus* epidemic.

**Figure 3:**
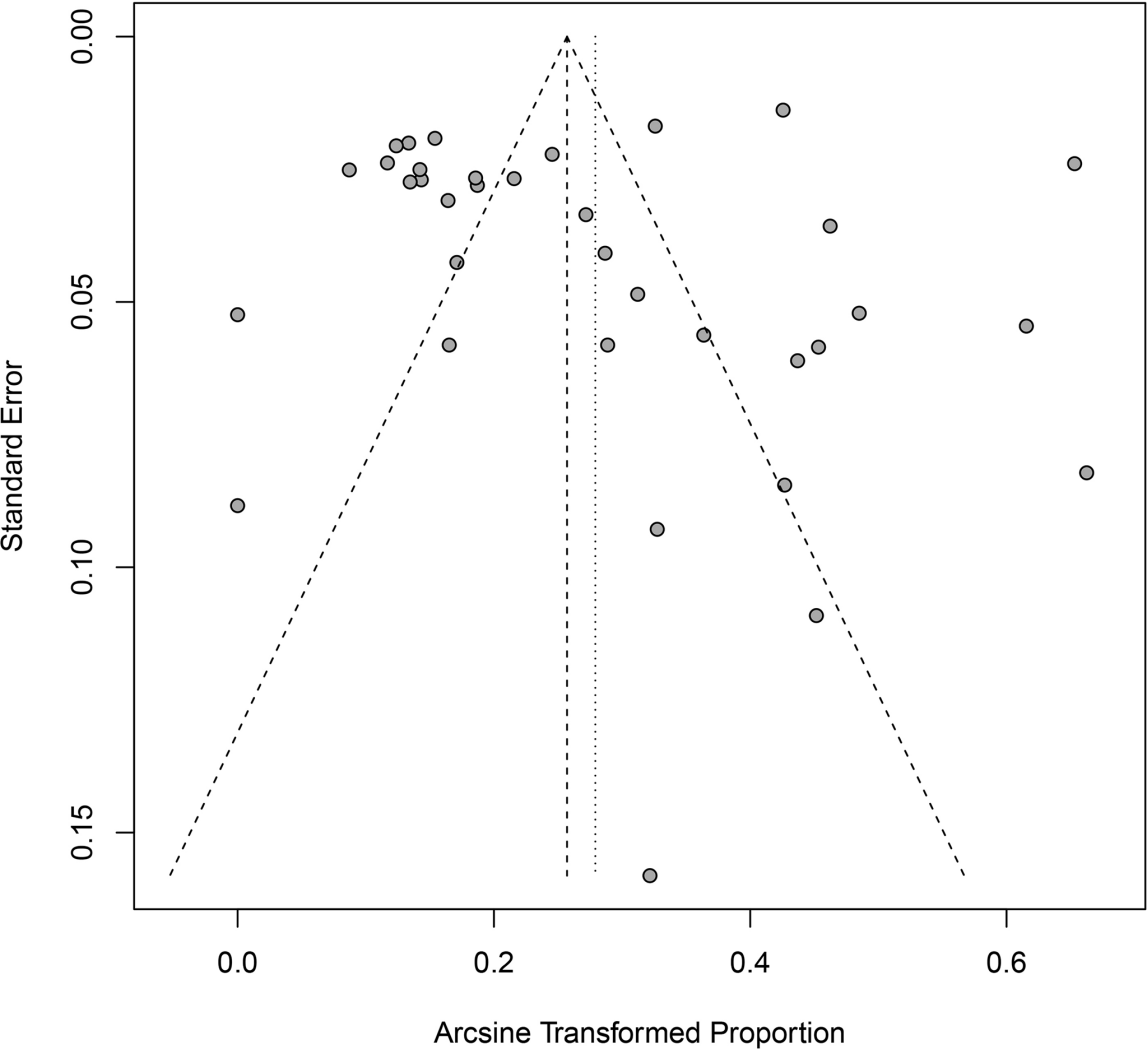
Funnel plot with pseudo 95% confidence interval for publication bias test.

**Figure 4:**
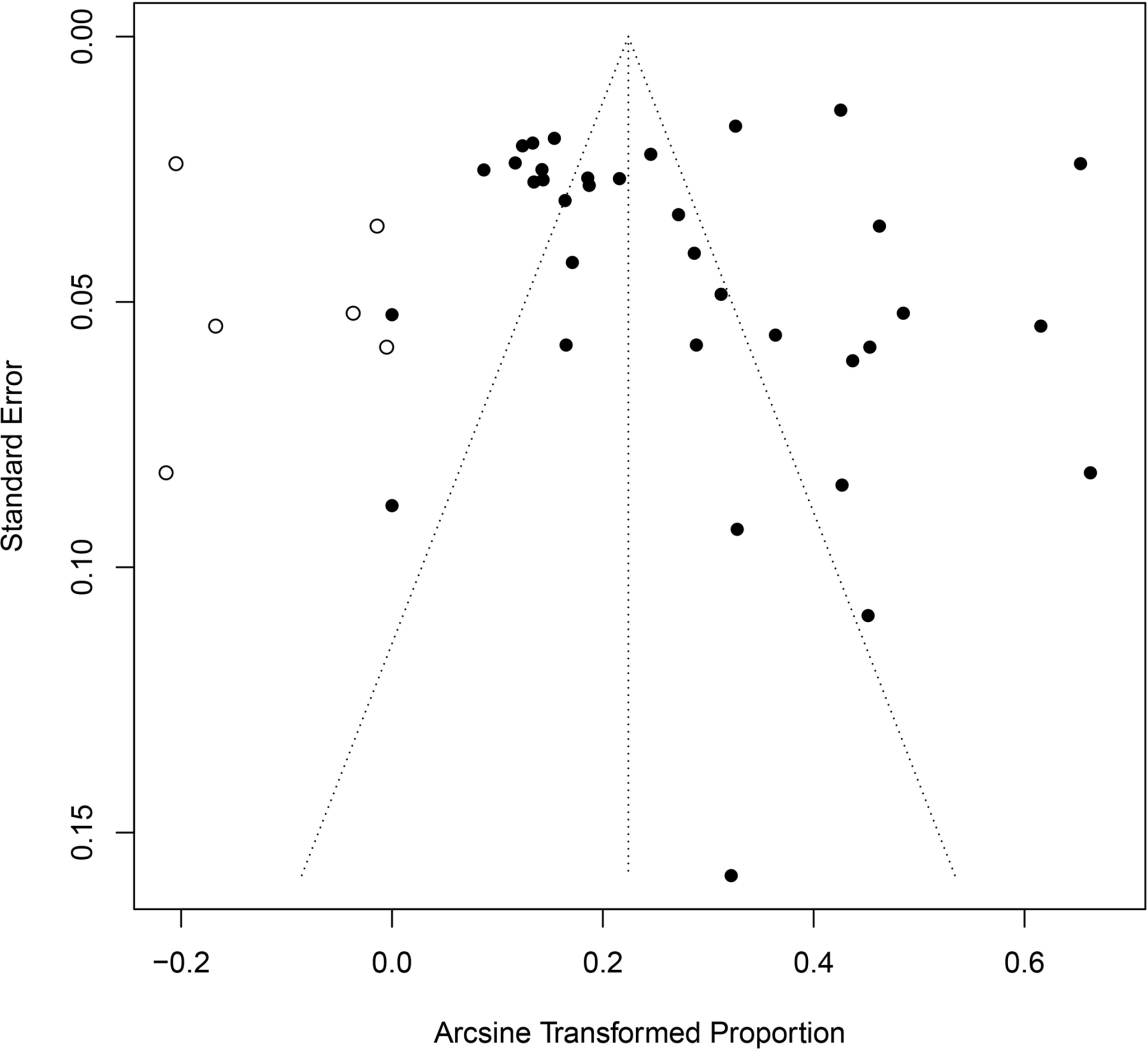
Shear complement graph and pseudo 95% confidence interval publication bias test.

**Figure 5:**
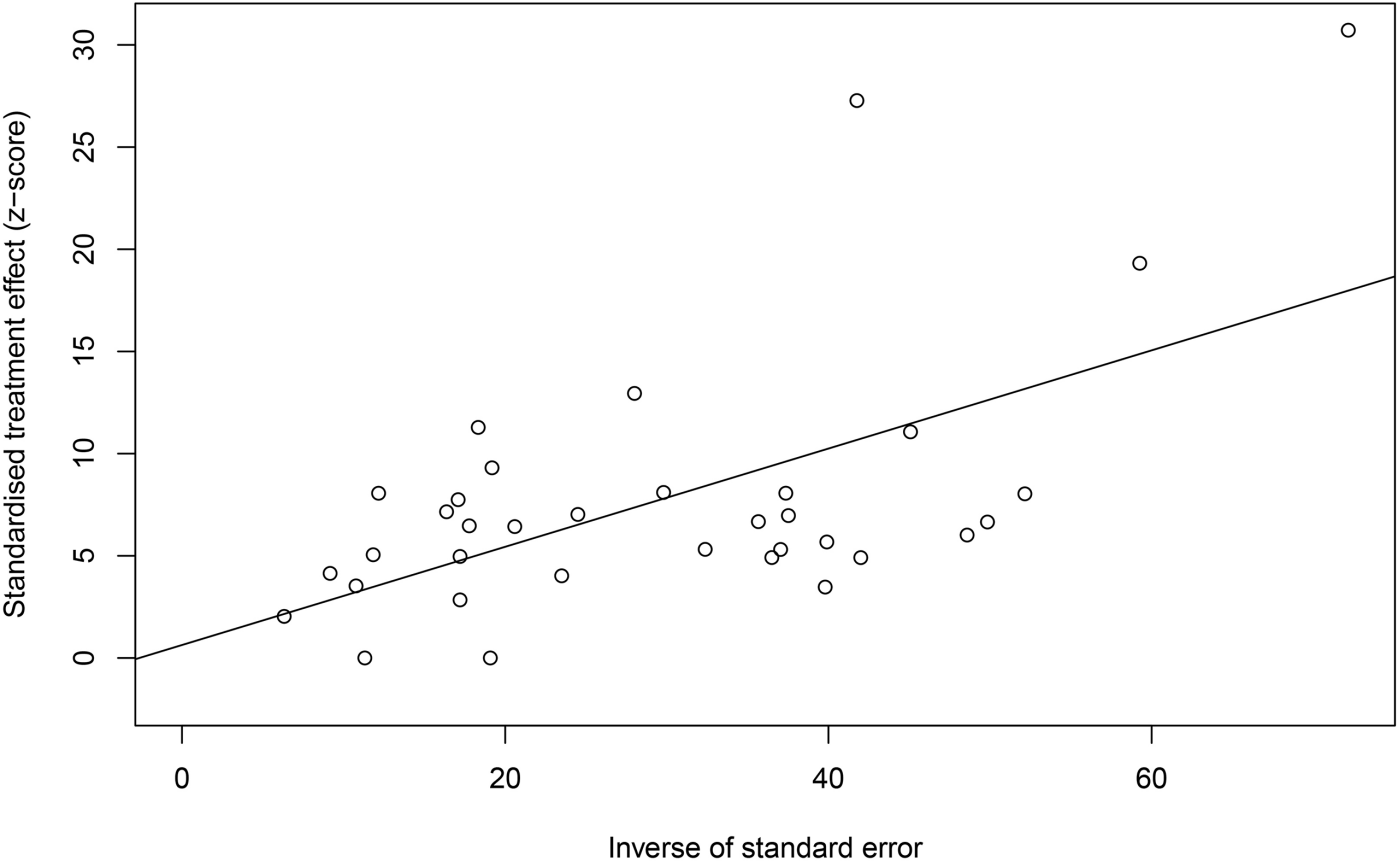
Egger’s test for publication bias.

**Figure 6:**
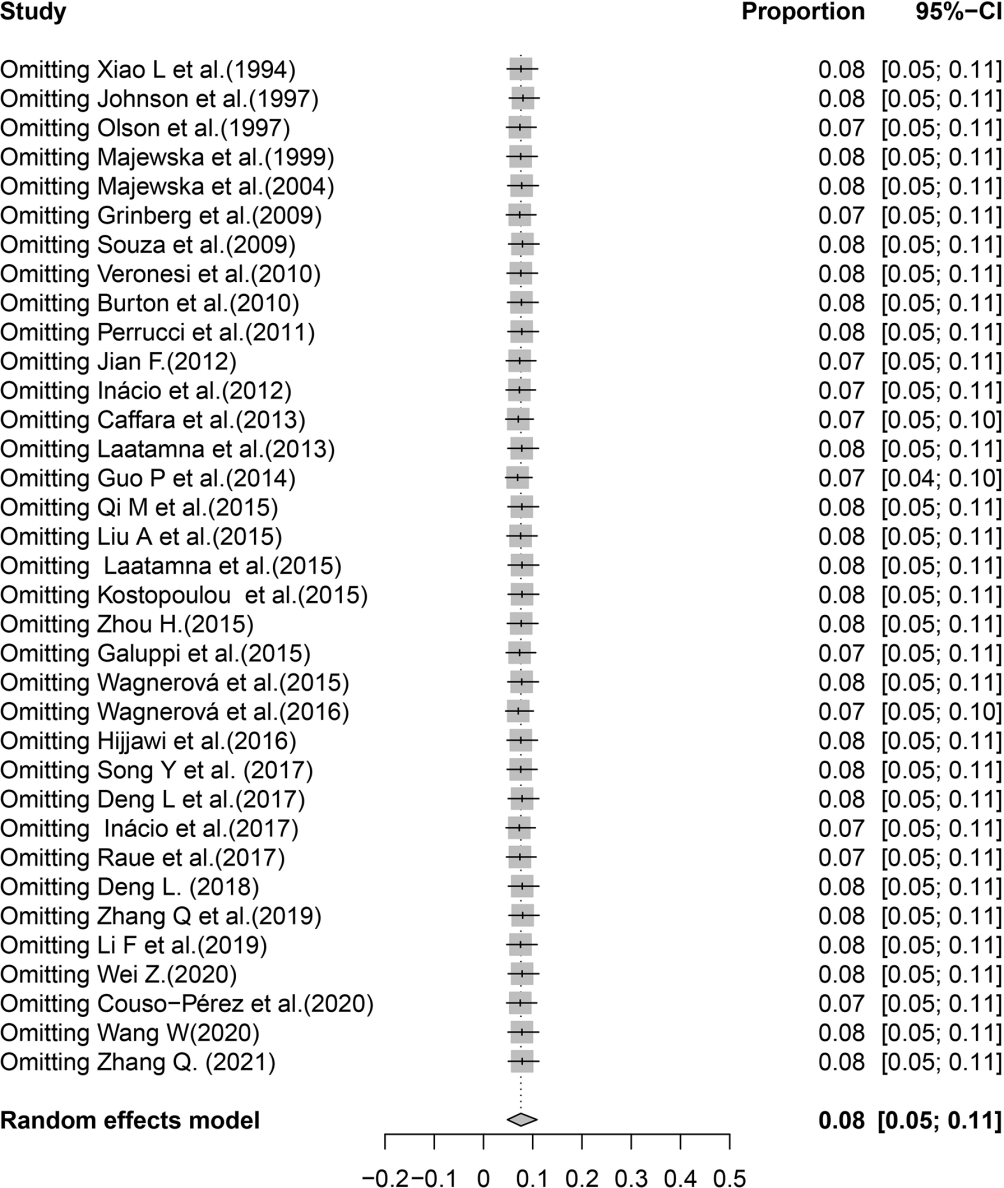
Sensitivity analysis.

**Figure 7:**
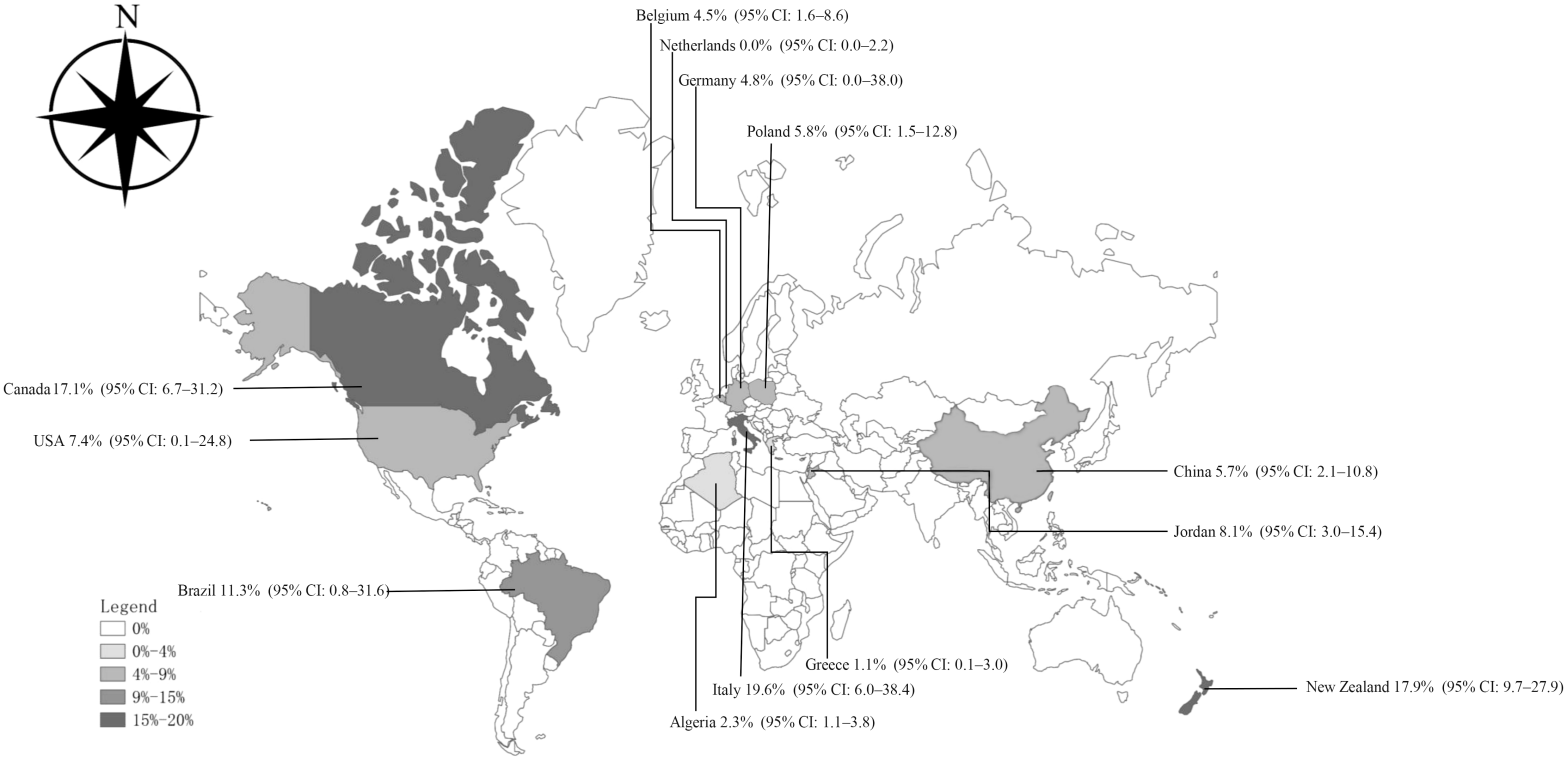
Map of *Cryptosporidium* prevalence in *Equus* worldwide.

**Figure 8:**
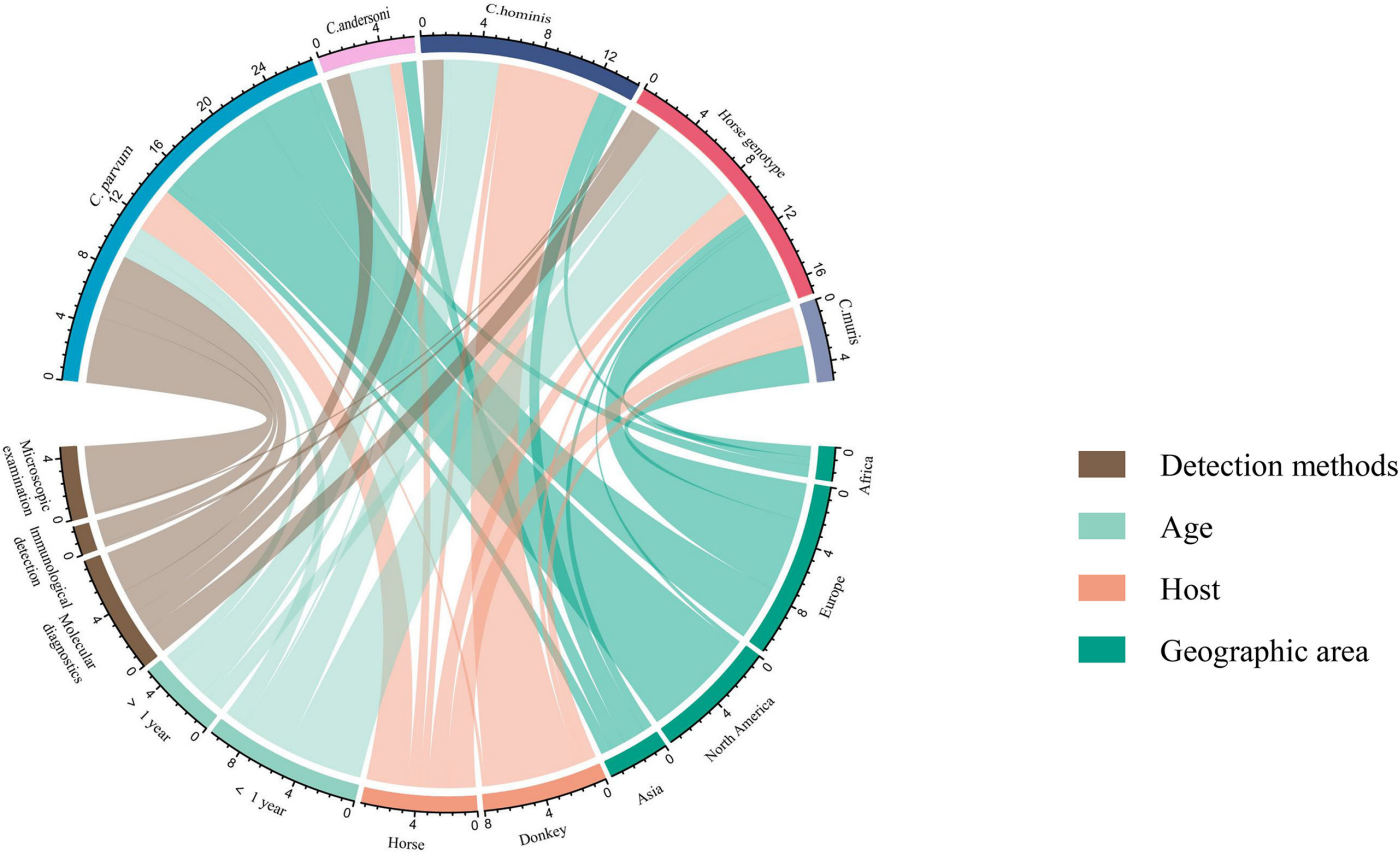
Distribution of *Cryptosporidium* species/genotypes.

In the included studies, the forest plot showed the degree of heterogeneity of all data (*χ* ^2^ = 0.0267, *I* ^2^ = 96.0%, *P* < 0.01) (**Figure 2**). According to the funnel chart, the distribution of points was observed to be incompletely symmetrical, which might be due to a publication bias (0.6344) or a small sample bias (**Figure 3**). Six supplementary studies were found in trim and fill analysis, which changed the aggregate estimate (**Figure 4**). The Egger’s test was used to assess the potential publication bias in the analysis with a *P*-value (0.7398) greater than 0.05, thus indicating that no publication bias was present in the data (**Figure 5**). The sensitivity test showed that the reorganized data were not significantly affected after excluding any study, and the results were consistent (**Figure 6**), which verified the rationality and reliability of this analysis. **Figure 7** is a map of *Cryptosporidium* prevalence in *Equus* worldwide. A chord diagram shows the relationship between the prevalence of *Cryptosporidium* in *Equus* species and epidemiological variables (stripe width indicates prevalence) (**Figure 8**). *C. parvum* genotype had a larger proportion in North America in the geographic area grouping. Genotype *C. hominis* accounted for a large proportion of donkey in the host group. Genotype *C. andersoni* accounted for more than 1- year-old in the age group.

### Meta-analysis of *Equus Cryptosporidium* worldwide

According to the data obtained from the selected articles, the global combined prevalence of *Equus Cryptosporidium* infection was 7.59% (95% CI: 4.86-10.87) (**Table 3**). Before 2008, the prevalence of *Cryptosporidium* in *Equus* was 10.77% (95% CI: 3.92-20.32), which was significantly higher than that in other time periods (*P* < 0.01). The highest positive rate of *Equus Cryptosporidium* was 17.91% (95% CI: 9.73-27.92) in Oceania. Among the detection methods, microscopic examination had the highest rate of 10.52% (95% CI: 4.99-17.19). The prevalence of *Cryptosporidium* in *Equus* ≤ 1-year-old was 11.06% (95% CI: 6.13-17.22), which was significantly higher than in other age groups (*P* < 0.01). The highest prevalence of *Cryptosporidium* in female *Equus* was 2.60% (95% CI: 0.78-5.44). The highest prevalence of *Cryptosporidium* among the rearing method groups was 7.86% (95% CI: 4.39-12.22) for collectivized breeding. Among the host groups, mules had the highest rate of 20.00% (95% CI: 10.20-32.09). The highest prevalence of *Equus Cryptosporidium* was 19.58% (95% CI: 6.03–38.43) in Italy, which was significantly higher than that in other countries (**Table 4**). The highest genotype prevalence of *Cryptosporidium* in *Equus* was *C. muris* (53.55%; 95% CI: 11.65-92.51), followed by *C. hominis*, with a rate of 43.94% (95% CI: 23.11–65.95) (**Table 5**).

**Table 4:** Concentrated prevalence of equine *Cryptosporidium* in different countries.

Variable	Category	No. studies	No. examined	No. positive	% (95% CI*)
Country	Algeria	2	481	11	2.27% (1.13–3.79)
	America	4	746	60	7.37% (0.08–24.78)
	Belgium	1	134	6	4.48% (1.64–8.61)
	Brazil	3	684	62	11.28% (0.76–31.64)
	Canada	1	35	6	17.14% (6.67–31.19)
	China	13	6122	562	5.65% (2.08–10.83)
	Germany	2	51	4	4.84% (0.00–38.02)
	Greece	1	190	2	1.05% (0.10–2.99)
	Italy	3	260	40	19.58% (6.03–38.43)
	Netherlands	1	44	0	0.00% (0.00–2.17)
	New Zealand	1	67	12	17.91% (9.73–27.92)
	Poland	2	424	21	5.81% (1.47–12.76)
	Jordan	1	74	6	8.11% (3.03–15.36)

**Table 5:** The prevalence of *Cryptosporidium* in different genotypes.

Variable	Category	No. studies	No. examined	No. positive	% (95% CI*)
Genotype	*C. parvum*	17	348	80	31.95% (15.53–51.11)
	*C. andersoni*	5	34	12	38.83% (12.50–69.36)
	*C. hominis*	8	165	103	43.94% (23.11–65.95)
	*C. muris*	2	19	11	53.55% (11.65–92.51)
	*Horse genotype*	10	245	41	29.24% (9.47–54.44)

Among the analyzed geographical factors, the positive rate of *Cryptosporidium* in *Equus* at northern latitude < 30° was the highest (8.77%; 95% CI: 2.88-17.44). The positive rate of *Cryptosporidium* in *Equus* with longitude > 100° was the highest (9.03%, 95% CI: 3.15-17.53). The information for other geographical latitude subgroup analyses included precipitation range (≥ 500 mm; 15.63%; 95% CI: 8.60-24.28), temperature range (≥ 10°C; 8.04%, 95% CI: 3.30-14.60), humidity range (70%-80%; 22.69%; 95% CI: 13.76-33.10) and altitude range (< 50 m; 6.88%; 95% CI: 2.70-12.78). According to the analysis of climatic factors, we found that tropical climate had the highest positive rate of *Equus Cryptosporidium* (16.46%; 95% CI: 0.48-48.11; **Table 6**).

**Table 6:** A subgroup analysis of the prevalence of *Cryptosporidium* in equine genus by geographical location, climate and other variables.

Variable	Category	No. studies	No. examined	No. positive	% (95% CI*)	Heterogeneity				Univariate meta-regression	
						*χ^2^*	*P*-value		*I^2^* (%)	*P*-value*	Coefficient (95% CI)
Latitude	< 30°	7	794	45	8.77% (2.88-17.44)	70.94	<0.01	91.5		0.1377	-0.1346(-0.3122 to 0.0431)
	30°-35°	3	809	56	3.67% (0.02-13.11)	60.19	<0.01	96.7			
	35°-40°	7	2357	213	2.70% (0.10-8.61)	281.98	<0.01	97.9			
	40°-45°	7	1283	52	5.17% (0.74-13.23)	70.21	<0.01	91.5			
	> 45°	8	1597	121	7.45% (3.77-12.26)	79.96	<0.01	91.2			
Longitude	< 50°	7	1383	66	5.62% (1.27-12.79)	76.48	<0.01	92.2		0.2230	-0.1085 (-0.2830 to 0.0660)
	50°-100°	6	2108	39	1.75% (0.7-3.26)	17.72	<0.01	71.8	
	> 100°	9	3282	370	9.03% (3.15-17.53)	305.79	<0.01	97.4	
Altitude(m)	< 50	15	3717	372	6.88% (2.70-12.78)	365.84	<0.01	96.2		0.2367	-0.1096(-0.2913 to 0.0720)
	50-100	6	858	21	2.41% (0.07-7.87)	31.02	<0.01	83.9	
	100-150	8	1722	73	4.23% (1.08-9.32)	72.01	<0.01	90.3	
	> 150	6	543	21	4.94% (0.06-16.98)	63.07	<0.01	92.1	
Rainfall (mm)	< 500	3	620	20	1.71% (0.06-5.54)	56.80	<0.01	84.2		0.0004	-0.2751 (-0.4277 to 0.1225)
	≥ 500	2	1436	220	15.63% (8.60-24.28)	117.90	<0.01	93.2	
Temperature (°C)	< 10	14	744	32	3.95% (0.34-11.25)	97.30	<0.01	86.6		0.3395	0.0872 (-0.0918 to 0.2662)
	≥ 10	11	1599	217	8.04% (3.30-14.60)	173.21	<0.01	94.2	
Humidity (%)	40-50	3	183	8	1.26% (0.00-8.59)	6.95	0.03	71.2		0.0367	0.3927 (0.0243 to 0.7610)
	50-60	10	581	33	4.41% (0.07-14.98)	93.76	<0.01	90.4	
	60-70	8	661	50	5.02% (0.94-12.07)	69.96	<0.01	90.0	
	70-80	3	569	142	22.69% (13.76-33.10)	10.57	<0.01	81.1	
Climate	Plateau alpine climate	2	52	9	12.71% (0.00-78.20)	26.62	<0.01	96.2		0.5059	-0.0998 (-0.3937 to 0.1942)
	Subtropical climate	15	2701	255	7.82% (3.27-14.10)	413.23	<0.01	96.6	
	Temperate climate	16	6251	455	5.81% (3.61-8.49)	359.24	<0.01	94.7	
	Tropical climate	3	567	55	16.46% (0.48-48.11)	123.02	<0.01	98.4	

CI*, Confidence interval; NA*, not applicable; P-value*: P < 0.05 is statistically significant.

## Discussion

*Cryptosporidium* is a waterborne pathogen that infects livestock, poultry, and companion animals, thus posing a great threat to public health (Gao et al., 2021). Many large *Cryptosporidium* outbreaks were caused by contamination of water sources with animal feces (Rodríguez et al., 2012). Cryptosporidiosis can cause slow growth of sick animals, extreme weight loss, decreased resistance, and huge losses to the animal husbandry (Han et al., 2020). In this study, a publication bias was observed according to the Egger’s test. Pass *I^2^* statistics results, the prevalence of *Cryptosporidium* in *Equus* species in the world was highly heterogeneous in the eligible studies, which may be caused by differences in detection methods, age, gender, geographic factors, and countries.

The prevalence of *Cryptosporidium* in female *Equus* was identified to be higher than that in male *Equus*. This is probably due to a weaker body resistance of female *Equus* than males, especially after giving birth, and more susceptible to *Cryptosporidium* infection (Chen et al., 2013). The mare is considered to be a carrier of *Cryptosporidium*, and the young animals could be infected with *Cryptosporidium* by sharing a pasture or barn with the foals (Qi and Zhang, 2018). The eggs of *Cryptosporidium* in infected female *Equus* have no obvious shedding phenomenon, but the amount of shedding during the perinatal period increases (Skerrett and Holland, 2001). This significantly increases the probability of young animals being infected with *Cryptosporidium*.

In this study, the prevalence of *Cryptosporidium* in *Equus* of ≤ 1 year was higher than that of *Equus* > 1 year, which is basically in line with the previous reports (Langkjaer et al., 2007; Wang, 2020). The maternal antibodies in the young animals will lose protective effects in 2-6 months, resulting in a decreased resistance of the young animals (Lu et al., 2008). In addition, the weaning stress response in young animals will lead to changes at hormone levels and immune function, thus causing immune system suppression, and thereby increasing the possibility of being infected with *Cryptosporidium* (Ren et al., 2018). Therefore, the breeding of dams and cubs needs to be strengthened to improve the resistance of animals, and the dams can be isolated during the breeding period.

Poor sanitary conditions increase the infection risk of *Cryptosporidium* in animals and expand the spread of *Cryptosporidium* species (Gao et al., 2021). Our study of the rearing group showed that mules and donkeys had higher rates of *Cryptosporidium* infection than horses. The living environment of mules and donkeys is relatively complicated, and they usually suffer from poor harness, lack of veterinary care, improper nutrition, and low status and value, in spite of their usefulness (Davis, 2019). Meanwhile, the feces cannot be cleaned in time, which is more likely to cause the transmission and infection of *Cryptosporidium* in animals (Jian, 2012). An analysis of the breeding environment also showed that the prevalence of *Cryptosporidium* was higher in *Equus* than that farmed on scale breeding. The high stocking densities of large-scale farming might result in high rates of *Cryptosporidium* infection in high-density captive equines. This may be due to the facts that more oocysts are scattered in high-density pens and the transmission speed is fast (Wang et al., 2008). Therefore, we recommend reducing stocking density and enhancing the animal welfare of donkeys and mules.

The investigation of sampling year in the selected articles showed that the prevalence of *Cryptosporidium* in *Equus* animals before 2012 was higher. In 2008, a worldwide economic crisis occurred and started to recover until 2010 (Zhang, 2018). The sluggish world economy may make people slack in *Equus* breeding, which may be one of the reasons for the high prevalence of *Cryptosporidium*. After 2013, the world economy gradually recovered, and many countries began to pay more attention to the animal husbandry. The gradually increased animal welfare may contributed to the lower prevalence of *Cryptosporidium* in *Equus*.

In the continent and country groups, the highest positive rate of *Cryptosporidium* was found in Oceania and the lowest was in Africa. Among them, New Zealand in Oceania had the highest positive rate, and Algeria in Africa had the lowest positive rate. New Zealand has a temperate maritime climate, with a warm and humid climate throughout the year, and its long coastline and abundant water resources make it more suitable for *Cryptosporidium* to survive. The frequent rainfall may lead to the transmission of *Cryptosporidium* in animal feces from fields to surface waters, thus resulting in a higher prevalence of *Cryptosporidium* in this genus of horses (Sterk et al., 2016). In contrast, the Algerian coast has a Mediterranean climate, with high temperature and rainy winter, while the central and southern parts have a savanna and tropical desert climate, which is dry and rainy, with cold winter and hot summer. The increase of temperature will reduce the life activity of the worms on the land surface (Moriarty et al., 2012). Therefore, the prevalence of *Cryptosporidium* in *Equus* in Africa is low.

In the geographical groups, the positive rate of *Cryptosporidium* was the highest in regions with longitude > 100° and latitude < 30°. These two factors are mainly concentrated in the central and southern parts of China. The central and southern parts of China belong to the temperate monsoon climate and the subtropical monsoon climate. The rainfall types of these two climates are high temperature and rainy in summer, low temperature and less rain in winter (Wang, 2009). Meanwhile, the positive rate of *Cryptosporidium* in *Equus* that live in areas with humidity 70%-80%, rainfall > 500 mm, temperature ≥ 10°C, and altitude < 50 m was higher than that in other regions. High rainfall will increase the pollution of water sources, and *Cryptosporidium* is mainly transmitted through water sources, thereby increasing the prevalence of *Cryptosporidium* (Young et al., 2015). As altitude decreases, the temperature gradually increases (Zhu et al., 2013). Suitable temperature and hot and humid weather are the reasons for an increase in the prevalence of *Cryptosporidium*. This is in line with previous reports (Burton et al., 2010; Lv et al., 2021).

The genotypes of *Cryptosporidium* in infected *Equus* were analyzed. The main genotypes of *Equus Cryptosporidium* were *C. parvum*, *horse genotype*, *C. andersoni, C. muris, C. hominis*, and *C. cuniculus* (Wang, 2015; Wagnerová et al., 2016; Deng, 2018; Zhang, 2021). Among them, *C. parvum, C. andersoni, and C. hominis* are the genotypes that infect humans. In this study, the positive rates of *C. muris* and *C. hominis* genotypes were higher. Therefore, strengthening the feeding, management, and control of *Equus Cryptosporidium* are of great importance to the public safety of humans and animals.

The included articles mainly employed three types of detection methods, including molecular detection, immunological detection, and microscopic detection. Molecular examination is mainly based on PCR. Immunological tests include IFAT, DAF, and ELISA. Among them, the positive rate detected by IFAT technology is the highest, followed by microscopy and PCR. The microscopic detection of *Cryptosporidium* is easily operated and has a low cost but may lead to misdiagnosis and increase the probability of false positives (Robinson and Chalmers, 2020a). For immunological detection, although ELISA has the advantages of high sensitivity and specificity, it cannot perform species identification (Mittal et al., 2014). IFAT is prone to false positive, thus resulting in a high positive rate (Robinson et al., 2020b). The PCR detection method have high sensitivity and specificity than that of microscopy, has become the optimal method for *Cryptosporidium* detection (Zhang et al., 2017). It is recommended to use PCR method to detect *Cryptosporidium* in epidemiological investigations.

These results reflect the prevalence of *Cryptosporidium* in *Equus* to a certain extent around the world. In this meta-analysis, the reasons for losing points in some studies are: (1) less than 3 risk factors; (2) limited samples; (3) unclear sampling year; and (4) absence of specific sampling location. It is recommended that researchers should take more samples and explore more influencing factors to provide more data that support for the prevention and treatment of *Cryptosporidium* infection in *Equus*.

The advantages of this study are rigorous method and lots of risk factors. The publication bias was tested by using funnel chart and more accurate Egger’s test. The subgroup analysis and regression analysis correctly identified the influence of heterogeneity on the results. The combination of multiple data analysis methods made our findings reasonable. A comprehensive analysis of subgroups can replenish the previous articles and provide more complete data for follow-up research. However, this study also has some limitations. First, we searched in five databases, and the search strategy might have caused the omission of some researches. Second, the data in some subgroups, such as Italy in the city subgroup, are covered only by one article, which can lead to unstable outcomes.

## Concluding remarks

A systematic review and meta-analysis of 35 articles provided a comprehensive overview of the global epidemiology of *Equus Cryptosporidium.* The results showed that cryptosporidiosis was widespread in *Equus*, and cryptosporidiosis was prone to occur in the areas with warm climate. *Equus* under 1 year were more susceptible to *Cryptosporidium* infection. In view of the high incidence of cryptosporidiosis, correct prevention and control measures should be taken in time for specific age groups and regions. During the breeding process, good hygiene habits should be developed, regional prevention and control should be strengthened, and water pollution should be minimized.

## Data availability statement

The original contributions presented in the study are included in the article/Supplementary Material. Further inquiries can be directed to the corresponding authors.

## Ethics statement

Ethical review and approval was not required for the animal study.

## Author contributions

X-ML and H-LG: Data curation, Methodology, Supervision, Writing-review and editing. W-LY and X-XZ: Writing-review and editing. Y-JW, Xin-YW: Data curation, Resources, Software. MZ and Xia-YW: Data curation, Methodology, Visualization. JL and GL: Conceptualization, Supervision, Funding acquisition. All authors contributed to the article and approved the submitted version.

## Funding

This work was supported by the Research Foundation for Distinguished Scholars of Qingdao Agricultural University (grant no. 665-1120044 and 665-1122009).

## Conflict of interest

The authors declare that the research was conducted in the absence of any commercial or financial relationships that could be construed as a potential conflict of interest.

## Publisher’s note

All claims expressed in this article are solely those of the authors and do not necessarily represent those of their affiliated organizations, or those of the publisher, the editors and the reviewers. Any product that may be evaluated in this article, or claim that may be made by its manufacturer, is not guaranteed or endorsed by the publisher.
